# ABAT Overexpression Protects Against Doxorubicin-Induced Cardiotoxicity by Preserving Mitochondrial Homeostasis

**DOI:** 10.3390/ijms27219994

**Published:** 2026-11-07

**Authors:** Peng Yang, Rong Yang, Jingjing Guo, Aonan Yu, Xinyu Liu, Mengxiao Zhang

**Affiliations:** 1 School of Pharmacy, Bengbu Medical University, Bengbu 233030, China; ypeng5454@163.com (P.Y.); sawazure@163.com (R.Y.); guojingjing0811@126.com (J.G.); y15055860069@163.com (A.Y.); 18355707020@163.com (X.L.); 2 Anhui Engineering Technology Research Center of Biochemical Pharmaceutical, Bengbu Medical University, Bengbu 233030, China

**Keywords:** ABAT, doxorubicin, cardiotoxicity, mitochondrial DNA, mitophagy, oxidative stress

## Abstract

Doxorubicin is a widely used chemotherapeutic agent whose clinical application is limited by dose-dependent cardiotoxicity, with mitochondrial dysfunction being a central pathological mechanism. Gamma-aminobutyric acid transaminase (ABAT) is a mitochondrial matrix enzyme implicated in mitochondrial DNA (mtDNA) maintenance through its role in the salvage synthesis of deoxyribonucleotides (dNTPs). However, whether ABAT exerts a protective role against doxorubicin-induced cardiotoxicity remains unknown. Here, we investigated the effect of ABAT overexpression on doxorubicin-induced cardiac injury and explored the underlying mechanisms. We found that ABAT expression and enzymatic activity were significantly reduced in the myocardium of doxorubicin-treated mice. Using cardiomyocyte-specific ABAT transgenic mice, we demonstrated that ABAT overexpression ameliorated doxorubicin-induced cardiac dysfunction, as evidenced by improved ejection fraction and fractional shortening, reduced serum levels of cardiac injury markers, and alleviated myocardial fibrosis and cardiomyocyte atrophy. Mechanistically, ABAT overexpression reversed doxorubicin-induced mtDNA depletion, restored mitochondrial respiratory chain complex activity, and improved oxygen consumption rate and ATP production, accompanied by reduced cellular and mitochondrial reactive oxygen species levels. Furthermore, doxorubicin triggered the initiation of mitophagy, as reflected by the accumulation of phosphatase and tensin homolog (PTEN)-induced kinase 1 (PINK1) and Parkin, but did not sustain autophagic degradation, as reflected by a markedly blunted bafilomycin A1-induced accumulation of microtubule-associated protein 1 light chain 3 beta II (LC3B-II) so that autophagic structures containing mitochondria accumulated; ABAT overexpression attenuated these changes, partially restoring the responsiveness of LC3B-II to bafilomycin A1. Complementary in vitro experiments using ABAT-overexpressing H9c2 cells confirmed that ABAT attenuated doxorubicin-induced lactate dehydrogenase (LDH) release, caspase-3 activation, and apoptosis. Taken together, our findings demonstrate that ABAT overexpression protects against doxorubicin-induced cardiotoxicity and is associated with preservation of mtDNA and mitochondrial function, reduced oxidative stress, and a reduced burden of damaged mitochondria. Given the reported antitumor properties of ABAT, these findings provide a basis for further investigating ABAT as a potential therapeutic target for mitigating doxorubicin-induced cardiotoxicity.

## 1. Introduction

Doxorubicin (DOX) is a widely used anthracycline antibiotic with potent antitumor activity against a broad spectrum of malignancies. Its clinical utility, however, is severely compromised by cumulative, dose-dependent cardiotoxicity, which can progress from subclinical myocardial injury to irreversible heart failure [1]. Despite decades of intensive investigation, the precise pathophysiological mechanisms underlying DOX-induced cardiotoxicity remain incompletely understood, and effective clinical interventions are still lacking. Elucidating these mechanisms and identifying novel therapeutic targets are therefore important clinical priorities.

Mitochondrial dysfunction is recognized as a central event in DOX-induced cardiotoxicity [2]. Owing to its high affinity for cardiolipin in the inner mitochondrial membrane, DOX preferentially accumulates in the mitochondria, where it impairs respiratory chain complex activity, disrupts mitochondrial dynamics, ultimately culminating in cardiomyocyte death [2]. Among these detrimental effects, depletion of mitochondrial DNA (mtDNA) is particularly consequential. Mammalian mtDNA encodes 13 essential subunits of the oxidative phosphorylation system, and its integrity is critical for maintaining mitochondrial bioenergetics [3]. Notably, DOX causes mtDNA depletion in cardiomyocytes, a phenomenon closely associated with mitochondrial dysfunction and myocardial injury [4].

4-Aminobutyrate aminotransferase (ABAT) is a mitochondrial matrix enzyme classically known for its role in γ-aminobutyric acid (GABA) catabolism. Intriguingly, recent studies have uncovered a non-canonical function of ABAT in mitochondrial nucleic acid metabolism. The enzyme catalyzes the conversion of deoxynucleoside diphosphates (dNDPs) to deoxynucleoside triphosphates (dNTPs), thereby supplying essential substrates for mtDNA salvage synthesis [5]. Germline loss-of-function mutations in *ABAT* have been linked to mtDNA depletion syndrome [5,6]. In our previous study of diabetic cardiomyopathy, ABAT was markedly downregulated in lipotoxic hearts, and cardiac-specific ABAT overexpression effectively restored mtDNA copy number, preserved mitochondrial function, and attenuated myocardial injury [7]. In preliminary experiments using a DOX model, we observed a similar reduction in ABAT in DOX-injured myocardium, raising the possibility that ABAT deficiency may be a contributor to DOX related cardiac injury. However, whether ABAT plays a protective role in DOX-induced cardiotoxicity, and through which mechanisms, remains entirely unexplored.

On this basis, we hypothesized that DOX-induced ABAT downregulation contributes to mtDNA depletion, leading to respiratory chain dysfunction, excessive oxidative stress, and aberrant mitophagy, ultimately culminating in cardiomyocyte death and cardiac functional decline. To test this hypothesis, we generated cardiomyocyte-specific ABAT transgenic mice and employed complementary in vitro models to systematically define the functional role and molecular underpinnings of ABAT in DOX cardiotoxicity. Our findings identify ABAT as an endogenous defender against DOX-induced myocardial injury and highlight its potential as a therapeutically tractable target for cardioprotection in patients receiving anthracycline chemotherapy.

## 2. Results

### 2.1. DOX Treatment Down-Regulates ABAT in Mouse Hearts

To investigate the effect of doxorubicin on ABAT level in cardiac tissue, we established a mouse model of DOX-induced cardiotoxicity. Quantitative PCR analysis revealed a significant reduction in ABAT mRNA levels in the hearts of DOX-treated mice compared to controls (Figure 1A). The enzymatic activity of ABAT was significantly reduced in the hearts of DOX-treated mice (Figure 1B). Subsequently, we observed a marked decrease in ABAT protein expression (Figure 1C,D). The identity of the ABAT band was further confirmed by an independent knockdown experiment (Supplementary Figure S1). These results confirmed that DOX treatment leads to downregulation of ABAT in mouse hearts.

### 2.2. Cardiomyocyte-Specific ABAT Overexpression Attenuates DOX-Induced Cardiac Dysfunction and Myocardial Injury

Since ABAT expression is markedly reduced in the hearts of DOX-treated mice, we sought to determine the functional significance of ABAT in DOX-induced cardiotoxicity. To this end, transgenic mice with cardiac-specific ABAT overexpression (TG-ABAT) were generated and validated by Western Blot (Figure 2A and Figure S2). To evaluate the impact of ABAT on DOX-induced cardiac injury, TG-ABAT mice and their wild-type (WT) littermates were intraperitoneally injected with DOX to establish a myocardial injury model. Five days after DOX administration, WT mice exhibited significant reduction in heart weight (Figure 2B), accompanied by impaired cardiac function as evidenced by decreased ejection fraction (EF) and fractional shortening (FS) (Figure 2C–E, Table S1). In contrast, TG-ABAT mice showed marked attenuation of cardiac atrophy, along with significant preservation of EF and FS (Figure 2B–E). To further validate the cardioprotective effect of ABAT, serum biomarkers of myocardial injury were assessed. DOX treatment significantly elevated circulating levels of creatine kinase-MB (CK-MB), cardiac troponin I (cTnI), lactate dehydrogenase (LDH), and aspartate aminotransferase (AST) in WT mice, whereas these increases were substantially blunted in TG-ABAT mice (Figure 2F–I). Collectively, these findings demonstrate that cardiomyocyte-specific overexpression of ABAT effectively alleviates DOX-induced myocardial injury and preserves cardiac function.

### 2.3. Overexpression of ABAT Ameliorates Doxorubicin-Induced Myocardial Structural Alterations and Mitigates Oxidative Stress in Mice

To further characterize the structural and biochemical alterations underlying DOX-induced cardiac injury, myocardial tissues were subjected to histological staining and oxidative damage assessment. Hematoxylin and eosin (H&E) staining revealed that DOX-treated WT mice exhibited pronounced myocardial disorganization, characterized by disrupted cardiomyocyte alignment, fiber fracture, and inflammatory infiltration. Notably, evident cardiomyocyte edema was observed, presenting as loose, pale-staining cytoplasm and vacuolation. These alterations were markedly attenuated in TG-ABAT mice (Figure 3A, Figures S3 and S4). In line with this, Masson’s trichrome staining demonstrated significant interstitial collagen deposition in WT mice following DOX exposure, which was markedly reduced in TG-ABAT mice, indicating alleviated cardiac fibrosis (Figure 3A,B). To further assess cardiomyocyte morphology, wheat germ agglutinin (WGA) staining was performed to evaluate cell size. Quantitative analysis showed that DOX treatment significantly reduced cardiomyocyte cross-sectional area in WT mice, whereas this effect was significantly reversed in TG-ABAT mice (Figure 3A,C). In line with these findings, the expression levels of cardiac stress markers atrial natriuretic peptide (ANP) and brain natriuretic peptide (BNP) were markedly elevated in WT mice after DOX administration, while their expression was significantly attenuated in TG-ABAT mice (Figure 3D,E). Collectively, these results indicate that ABAT overexpression effectively mitigates DOX-induced myocardial structural damage and pathological remodeling.

Given the well-established role of oxidative stress in DOX-induced cardiotoxicity, we next examined myocardial oxidative damage. DOX administration markedly increased protein carbonyl content and malondialdehyde (MDA) levels in WT mice, reflecting enhanced protein oxidation and lipid peroxidation, respectively (Figure 3F,G). Notably, these increases were significantly blunted in TG-ABAT mice. These findings demonstrate that cardiomyocyte-specific overexpression of ABAT not only alleviates DOX-induced myocardial structural damage but also suppresses oxidative injury in the heart.

### 2.4. ABAT Overexpression Attenuates DOX-Induced Cardiomyocyte Oxidative Stress and Apoptosis

To further determine whether ABAT directly protects cardiomyocytes against DOX-induced injury, H9c2 cardiomyocytes with ABAT overexpression were established, and the efficiency of ABAT upregulation was confirmed by Western blot analysis (Figure 4A). Following DOX stimulation, cell injury and apoptotic responses were evaluated. Compared with control cells, DOX exposure significantly increased LDH release in the culture medium, indicating enhanced cardiomyocyte injury, whereas ABAT overexpression markedly reduced LDH activity (Figure 4B). Similarly, DOX treatment induced a significant increase in cleaved-caspase-3 expression and caspase-3 activity, indicative of increased apoptosis. In contrast, ABAT overexpression substantially decreased cleaved-caspase-3 accumulation and caspase-3 activity following DOX exposure (Figure 4C–E), demonstrating that ABAT protects cardiomyocytes from DOX-induced apoptotic injury.

Given that mitochondrial oxidative stress is a critical contributor to DOX-induced cardiotoxicity, we next examined intracellular and mitochondrial ROS production. Flow cytometric analysis revealed that DOX treatment markedly increased intracellular ROS levels, whereas ABAT overexpression significantly attenuated ROS accumulation (Figure 4F,G). Furthermore, MitoSOX staining demonstrated enhanced mitochondrial superoxide generation in DOX-treated cardiomyocytes, which was significantly reduced by ABAT overexpression (Figure 4H,I). Collectively, these findings indicate that ABAT exerts a direct cardioprotective effect in cardiomyocytes by suppressing DOX-induced oxidative stress, mitochondrial ROS accumulation, and apoptosis.

### 2.5. ABAT Overexpression Preserves Mitochondrial Homeostasis in DOX-Treated Cardiomyocytes

Given that mitochondrial dysfunction is a key pathological mechanism underlying DOX-induced cardiotoxicity, and that ABAT has been reported to maintain mtDNA homeostasis via the mitochondrial salvage pathway, we next investigated whether ABAT modulates mitochondrial homeostasis under DOX stress. As shown in Figure 5A, DOX treatment significantly reduced mtDNA copy number, indicating impaired mitochondrial maintenance, whereas ABAT overexpression markedly restored mtDNA levels (Figure 5A). Consistently, calcein-AM staining revealed that DOX exposure induced pronounced opening of the mitochondrial permeability transition pore (mPTP), while ABAT overexpression effectively suppressed mPTP opening, suggesting improved mitochondrial membrane stability (Figure 5B). We further assessed mitochondrial functional capacity by examining respiratory chain activity and cellular bioenergetics. DOX treatment significantly decreased the activities of mitochondrial complexes I, III, and IV, whereas ABAT overexpression markedly reversed these impairments (Figure 5C–E). In parallel, DOX reduced the oxygen consumption rate (OCR) and increased the extracellular acidification rate (ECAR), accompanied by diminished ATP production, all of which were significantly alleviated by ABAT overexpression (Figure 5F–H). Collectively, these findings demonstrate that ABAT preserves mitochondrial integrity and bioenergetic capacity, thereby mitigating DOX-induced mitochondrial dysfunction.

### 2.6. Overexpression of ABAT Inhibits Doxorubicin-Induced Perturbation of Mitophagy in Cardiomyocytes

Since mtDNA depletion and mitochondrial dysfunction are potent triggers of mitophagy, and our data demonstrate that ABAT preserves mitochondrial homeostasis under DOX stress, we hypothesized that ABAT may exert its protective effects by modulating mitophagy. Transmission electron microscopy showed that DOX-treated cardiomyocytes contained more double-membrane structures enclosing mitochondrial profiles than vehicle-treated cells; these structures are referred to hereafter as autophagic structures containing mitochondria. ABAT overexpression reduced the number of these structures and alleviated mitochondrial ultrastructural damage (Figure 6A). Accumulation of such structures is by itself ambiguous, since it may reflect either increased initiation or impaired completion; we therefore assessed both arms of the pathway. Autophagic degradation was first examined by measuring flux with bafilomycin A1 (Baf A1) in the same four groups (Figure 6B). In vehicle-treated cells, Baf A1 produced a marked accumulation of LC3B-II in both Ad-Ctrl and Ad-ABAT cells, indicating active basal flux. In DOX-treated cells, the abundance of both LC3B-I and LC3B-II was markedly reduced, the LC3B-II/LC3B-I ratio was elevated, and Baf A1 produced only a modest additional accumulation of LC3B-II, far smaller than in vehicle-treated cells. Because flux is read out as the Baf A1-induced increment rather than as the steady-state level of LC3B-II, these data indicate that DOX compromises autophagic degradation. In DOX-treated cells overexpressing ABAT, the elevated LC3B-II/LC3B-I ratio was partially restored toward the vehicle level, and Baf A1 again produced a larger increment in LC3B-II than in DOX-treated control cells, indicating a partial recovery of the sensitivity of LC3B-II to Baf A1. This recovery was nevertheless incomplete, since the Baf A1-induced increment remained well below that of vehicle-treated cells, and the difference between Ad-ABAT and Ad-Ctrl cells under DOX did not reach statistical significance. ABAT overexpression therefore partially restores the autophagic degradation of LC3B-II without returning it to the control level.

We next examined PINK1 and Parkin, which report the initiation of mitophagy. In H9c2 cells, DOX significantly increased PINK1 and Parkin protein levels, and ABAT overexpression attenuated these increases (Figure 6C–E). The same pattern was observed in vivo, whereas the DOX-induced elevation of PINK1 and Parkin in myocardial tissue was reduced in the TG-ABAT mice (Figure 6F–H). These results indicate that mitophagy initiation was engaged rather than abolished under DOX stress, and ABAT overexpression reduces the burden of damaged mitochondria.

## 3. Discussion

In this study, we demonstrate that ABAT overexpression protects against DOX-induced cardiotoxicity. DOX exposure markedly reduced ABAT expression and enzymatic activity in the myocardium, whereas cardiac-specific ABAT overexpression alleviated cardiac dysfunction, myocardial injury, pathological remodeling and oxidative stress. Mechanistically, ABAT overexpression preserved mtDNA content and mitochondrial bioenergetic function, reduced mitochondrial oxidative stress, and limited the accumulation of damaged mitochondria under DOX stress, with partial restoration of autophagic degradation. These findings support a model in which DOX-induced suppression of ABAT contributes to the disruption of mitochondrial homeostasis and subsequent cardiac injury, whereas restoration of ABAT helps maintain mitochondrial homeostasis and protect the heart from DOX-induced damage.

Our initial observation that DOX downregulates myocardial ABAT is notable, as ABAT is not merely a GABA-catabolic enzyme but also a mitochondrial matrix protein with a significant role in mtDNA salvage synthesis. Given that mtDNA depletion is a well-documented driver of respiratory chain failure, oxidative stress, and cardiomyocyte death in DOX-related cardiac injury [8], we reasoned that reduced ABAT expression may contribute to the mitochondrial injury that characterizes DOX-induced cardiotoxicity. We found that ABAT overexpression attenuated DOX-induced mtDNA depletion and preserved mitochondrial respiratory complex activities, OCR, ECAR, and ATP production. Although these findings do not establish a direct role for ABAT in mtDNA replication or nucleotide salvage under DOX exposure, they support a potential association between increased ABAT expression and the preservation of mitochondrial homeostasis during cardiotoxic stress. However, because our study did not determine whether ABAT loss of function aggravates or directly induces cardiac injury, the significance of DOX-induced ABAT downregulation remains to be established.

DOX-induced mitochondrial dysfunction is a potent trigger of mitophagy. Since our data show that ABAT preserves mitochondrial homeostasis under DOX stress, we hypothesized that ABAT may exert its protective effect by modulating mitophagy. Our results indicate that DOX engages mitophagic initiation but fails to sustain its downstream completion. PINK1 and Parkin accumulated, indicating that the initiation machinery was activated rather than suppressed, yet the Baf A1-induced increment in LC3B-II was markedly blunted, and autophagic structures containing mitochondria accumulated on electron microscopy. This dissociation between initiation and degradation is consistent with prior evidence that DOX leaves autophagic induction largely intact but specifically impairs the fusion of autophagosomes with lysosomes, thereby reducing autolysosome formation and causing the accumulation of dysfunctional mitochondria [9]. Thus, under DOX stress, the limiting factor is not the initiation of mitophagy but the capacity to complete it, and PINK1/Parkin accumulation should be interpreted as a signal of mitochondrial damage and mitophagic initiation, not as evidence of increased mitophagic flux. ABAT overexpression attenuated PINK1/Parkin accumulation, reduced the number of autophagic structures, and partially restored both the LC3B-II/LC3B-I ratio and the Baf A1-induced increment in LC3B-II, although this recovery remained incomplete and did not reach statistical significance. ABAT therefore appears to act both by limiting the burden of damaged mitochondria and by partially restoring degradative capacity.

The role of PINK1/Parkin-mediated mitophagy in DOX cardiotoxicity is complex and context-dependent. On the one hand, appropriate PINK1/Parkin-mediated mitophagy can selectively remove damaged mitochondria and contribute to cardioprotection. For example, crocin-induced activation of PINK1-dependent mitophagy alleviated DOX cardiotoxicity, while inhibition of PINK1 or mitophagy abolished this protection [10]. On the other hand, excessive or maladaptive mitophagy may become detrimental under sustained DOX stress [11,12]. DOX has been shown to activate the PINK1/Parkin pathway and promote mitophagy, whereas pharmacological inhibition of mitophagy attenuates mitochondrial oxidative stress and preserves mitochondrial function in cardiomyocytes [13]. Consistently, inhibition of the NEU1–Drp1 axis was reported to suppress PINK1 stabilization, Parkin recruitment, and excessive mitophagy, accompanied by reduced apoptosis and improved cardiac function following DOX exposure [14]. These divergent observations suggest that the biological consequence of mitophagy depends on its magnitude, duration, and the integrity of the overall autophagic process [12]. Our data reconcile these findings by showing that the defect lies in the coupling between initiation and completion rather than in initiation alone. This distinction also changes the interpretation of ABAT’s protective effect. Rather than suppressing a protective response, ABAT reduces the mitochondrial damage that initiates mitophagy, so that fewer damaged mitochondria require processing and fewer incompletely processed autophagic structures accumulate, while the degradative step itself is partially restored. Thus, our findings favor the interpretation that ABAT helps restore mitochondrial turnover toward homeostasis under DOX stress, although whether this change in mitophagy is causally required for ABAT-mediated cardioprotection remains to be determined.

Previous studies of vitamin B6 and its derivatives provide additional biological context for our findings. Vitamin B6 supplementation and the vitamin B6 derivative pyridoxamine have been reported to attenuate DOX-induced cardiac dysfunction, oxidative stress, fibrosis, and apoptosis [15]. Pyridoxamine has also been reported to preserve the anti-tumor activity of DOX in a tumor model [16]. These findings are particularly relevant because ABAT is a pyridoxal 5′-phosphate (PLP)-dependent enzyme [17]. Although these studies do not demonstrate that the cardioprotective effects of vitamin B6 derivatives are mediated through ABAT, the convergence of these independent observations is noteworthy. These studies raise the possibility that PLP-dependent metabolic pathways may contribute to the cardiac response to DOX and that ABAT may represent one component of this protective metabolic network. Whether vitamin B6 availability or pyridoxamine treatment modulates ABAT activity or expression, and whether this contributes to their cardioprotective effects, warrant further investigation.

From a translational perspective, ABAT may represent a promising candidate for cardioprotective intervention in patients receiving anthracycline chemotherapy. An important consideration in cardio-oncology is whether cardioprotective strategies interfere with antitumor efficacy. This issue may be particularly relevant to ABAT because previous studies have reported reduced ABAT expression in several malignancies and suggested tumor-suppressive effects of ABAT restoration [18,19,20]. These observations provide a rationale for further investigating ABAT-based strategies. Nevertheless, whether ABAT augmentation can protect the heart while preserving or enhancing the antitumor efficacy of DOX remains to be determined.

## 4. Materials and Methods

### 4.1. Animals

Male C57BL/6J mice were provided by Hangzhou Ziyuan Laboratory Animal Science and Technology Co., Ltd. (Hangzhou, China; Laboratory Animal Production License No.: SCXK (Zhejiang) 2024–0004). Transgenic mice with cardiac-specific expression of the tetracycline transactivator (Tg-tTA) and transgenic mice harboring a tetracycline response element (TRE)-driven human *ABAT* gene (Tg-ABAT) were kindly provided by the research group of Professor Tianqing Peng at Soochow University. All mice were housed in a specific pathogen-free (SPF) barrier facility at Bengbu Medical University and had ad libitum access to sterilized feed and drinking water. The animal experimental protocols were reviewed and approved by the Laboratory Animal Management and Ethics Committee of Bengbu Medical College (protocol No. 2023–171, approval date: 13 March 2023).

The Tg-ABAT line uses a Tet-off system. Briefly, full-length human ABAT cDNA is driven by a tTA-responsive promoter, with cardiomyocyte-specific tTA expression directed by the α-myosin heavy chain (α-MHC) promoter. To generate transgenic mice with cardiomyocyte specific overexpression of ABAT, Tg-ABAT mice were crossed with Tg-tTA mice, and offspring were genotyped by tail-tip PCR. Mice double-positive for Tg-ABAT and tTA (Tg-ABAT/tTA) were defined as cardiac-specific ABAT-overexpressing mice (TG-ABAT). Mice positive for only Tg-ABAT or tTA, as well as double-negative mice, were collectively classified as wild-type (WT) mice because they lacked the ABAT overexpression phenotype [7].

### 4.2. Establishment of the In Vivo Model of DOX-Induced Cardiotoxicity

To establish an in vivo model of doxorubicin-induced cardiotoxicity, mice aged 6–8 weeks were treated with a single dose of DOX (15 mg/kg, i.p., Solarbio Life Sciences, Beijing, China). Sham mice received the same volume of sterile saline. After 5 days of DOX administration, mice were sacrificed with hearts and serum harvested for subsequent experiments. For molecular and biochemical assays, the atria and right ventricular free wall were removed, and the left ventricular tissue was snap-frozen and stored at −80 °C. For histological analyses, the apical half of the heart was fixed, embedded, and sectioned; full transverse sections of the ventricles from the cut surface were used for histological staining.

### 4.3. Assessment of Cardiac Function

Cardiac function was assessed by ultrasound examination after modeling following standardized protocols. Briefly, mice were anesthetized by inhalation with 2% isoflurane (RWD Life Science Co., Ltd., Shenzhen, China) and positioned supine on a temperature-controlled platform. Echocardiography was performed using a MS-400 high-frequency transducer attached to the VEVO 2100 ultrasound system (Fujifilm VisualSonics, Inc., Toronto, ON, Canada). Left ventricular internal diameters at end-systole and end-diastole were acquired at the papillary muscle level, and left ventricular ejection fraction (EF) and left ventricular fractional shortening (FS) were calculated.

### 4.4. Histopathological Examination of Cardiac Tissue

Mouse heart tissues were fixed in 4% paraformaldehyde, dehydrated through a graded ethanol series, cleared in xylene, and embedded in paraffin. Serial sections (4 μm) were prepared and subjected to hematoxylin and eosin (H&E), wheat germ agglutinin (WGA), and Masson’s trichrome staining according to the instructions of the corresponding kits (Servicebio, Wuhan, China). After staining, the sections were scanned using a slide scanner to acquire images. Cardiomyocyte cross-sectional area (CSA) in WGA-stained sections was used to represent cell size, and the collagen volume fraction in Masson-stained sections was used to represent the degree of fibrosis. All images were analyzed using ImageJ (version 1.54f, National Institutes of Health, Bethesda, MD, USA).

### 4.5. Detection of Serum Cardiac Injury Markers

CK-MB and cTnI levels in serum were determined using commercial ELISA kits (Sangon Biotech, Shanghai, China) according to the manufacturer’s instructions. Serum LDH and AST levels were determined using commercial kits (Beyotime, Nanjing, China).

### 4.6. Oxidative Stress Assay

Malondialdehyde (MDA) and protein carbonyl levels in myocardial tissue lysates were determined using an MDA assay kit (Jiancheng, Nanjing, China) and a protein carbonyl colorimetric assay kit (Solarbio Life Sciences, Beijing, China), respectively, to assess oxidative damage. The production of reactive oxygen species (ROS) and mitochondrial superoxide in H9c2 cell was evaluated using an ROS assay kit (Beyotime, Nanjing, China) and a MitoSOX™ Red kit (Beyotime, Nanjing, China). All procedures were performed according to the manufacturers’ instructions.

### 4.7. Cell Culture

Rat cardiomyocytes (H9c2) were purchased from Wuhan Pricella Life Science & Technology Co., Ltd. (Wuhan, China) and authenticated by short tandem repeat (STR) profiling. The cells were cultured in DMEM basal medium supplemented with 10% fetal bovine serum, 100 U/mL penicillin, and 100 μg/mL streptomycin, and maintained in a humidified atmosphere with 5% CO_2_ at 37 °C.

To overexpress ABAT in vitro, H9c2 cells were infected with an adenoviral vector encoding human ABAT (Ad-ABAT, Hanheng Biotechnology, Shanghai, China) at 25 pfu/cell, and an empty adenoviral vector (Ad-Ctrl) served as the control.

To establish an in vitro model of DOX-induced cardiomyocyte injury, H9c2 cells were seeded into cell culture plates at a density of 60,000 cells/mL and cultured for 24 h. Subsequently, 500 nM DOX or normal saline was added, and the cells were cultured for an additional 24 h. After treatment, the cells were washed twice with PBS to remove dead cells and debris and then harvested for downstream assays.

### 4.8. Determination of Cell Viability

H9c2 cells were seeded into 96-well plates and treated with 500 nM DOX for 24 h. CCK-8 solution (APExBIO Technology LLC, Houston, TX, USA) was added to each well, and the plate was incubated for 1 h in 37 °C incubator. The absorbance was then measured at 450 nm using a BioTek Synergy HT microplate reader (BioTek Instruments, Inc., Winooski, VT, USA).

### 4.9. Assessment of ABAT Activity

Tissues were homogenized in lysis buffer (10 mM K_2_HPO_4_/KH_2_PO_4_, 0.13% Triton X-100, 0.1 mM glutathione, 0.1 mM pyridoxal phosphate, 1 mM Na_2_EDTA). After centrifugation at 12,000× *g* for 30 min at 4 °C, the supernatant containing 80 µg of total protein, or lysis buffer alone as a blank control, was mixed with 180 µL of assay buffer (100 mM potassium pyrophosphate, 5 mM α-ketoglutarate, 4 mM NAD^+^, 2 mM DTT, 0.1 mM PLP). Following a pre-incubation at 30 °C for 15 min, γ-aminobutyric acid (GABA) was added to the mixture to a final concentration of 10 mM. After incubation for 30 min, the absorbance at 340 nm was measured.

### 4.10. Detection of Mitochondrial Permeability Transition Pore (mPTP) Opening

The mPTP opening of H9c2 cells was detected using an mPTP fluorescence assay kit (Beyotime, Nanjing, China) according to the manufacturer’s instructions. Briefly, cells were washed twice with pre-warmed PBS and treated with Calcein AM staining solution and incubated at 37 °C for 45 min in the dark. After incubation, the solution was replaced with fresh culture medium pre-warmed to 37 °C, and the cells were further incubated for 30 min at 37 °C. Finally, nuclei were stained with Hoechst 33342 for 10 min. After incubation, the cells were washed twice with PBS and observed using a Zeiss Observer Z1 fully motorized inverted fluorescence microscope (Carl Zeiss, Oberkochen, Germany).

### 4.11. Monitoring Autophagic Flux in Cells

Autophagic flux was assessed with bafilomycin A1 (Baf A1), an inhibitor of the vacuolar H^+^-ATPase that blocks autophagosome–lysosome fusion and lysosomal degradation. H9c2 cells infected with Ad-ABAT or Ad-Ctrl were treated with DOX (500 nM) or vehicle for 24 h; Baf A1 (100 nM) or an equal volume of vehicle was added during the last 4 h. Cells were then lysed and LC3B-I/II was detected by Western blotting. The flux was expressed as the Baf A1-induced increment in LC3B-II (LC3B-II normalized to the loading control).

### 4.12. Extracellular OCR Detection

Cellular oxygen consumption was measured using a Extracellular OCR Plate Assay Kit (Dojindo Molecular Technologies, Kumamoto, Japan). Following the manufacturer’s instructions, the plate was placed into a fluorescence plate reader preheated to 37 °C and incubated for 30 min. Then, one drop of mineral oil was immediately added to each well, and the plate was further incubated in the fluorescence plate reader at 37 °C for 5 min. The reader was set to continuous reading mode, with measurements taken every 10 min for a total duration of 200 min (excitation: 500 nm, emission: 650 nm, bottom reading mode). The final data were used to calculate the OCR values.

### 4.13. Extracellular Acidification Rate (ECAR) Detection

Cellular ECAR was measured using a ECAR Fluorometric Assay Kit (Elabscience Biotechnology Co., Ltd., Wuhan, China). Following the kit instructions, the fluorescence intensity of each well was measured using a fluorescence microplate reader at excitation and emission wavelengths of 490 nm and 535 nm, respectively. Readings were taken every 2–3 min over a total period of approximately 100–120 min. A plot of fluorescence intensity versus time was generated, and the linear segment of the curve was used to calculate the ECAR.

### 4.14. Detection of Cellular ROS by Flow Cytometry

Intracellular reactive oxygen species (ROS) were detected using the 2′,7′-dichlorodihydrofluorescein diacetate (DCFH-DA) probe (Beyotime, Nanjing, China) by flow cytometry. Briefly, H9c2 cells were harvested, washed with PBS, and incubated with 10 μM DCFH-DA in serum-free medium at 37 °C for 20 min in the dark. After loading, the cells were washed twice with serum-free medium to remove extracellular probe and resuspended in PBS. Fluorescence was measured immediately on a flow cytometer in the FITC channel (excitation 488 nm, emission 525 nm), and the mean fluorescence intensity was quantified with the accompanying software.

### 4.15. Quantification of mRNA and mtDNA by Real-Time PCR

For mRNA determination, total RNA was extracted from heart tissue using the TRIzol reagent (Biosharp, Hefei, China) following the manufacturer’s instructions. Real-time reverse transcription-PCR was performed as previously described [21]. The sequences of primers are as follows: *Abat*, 5′-GGAGCATCGGAAGGTGATCG-3′ and 5′-CCCGCGTCCTGATTAGATGG-3′; *Anp*, 5′-CTGCTAGACCACCTGGAGGA-3′ and 5′-AAGCTGTTGCAGCCTAGTCC-3′; *Bnp*, 5′-GAGGTCACTCCTATCCTCTGG-3′ and 5′-GCCATTTCCTCCGACTTTTCTC-3′; *Gapdh*, 5′-CAGTGTTGGGGGCTGAGTTC-3′ and 5′-AAAGGGCATCCTGGGCTACA-3′.

For mtDNA quantification, total DNA was isolated using Animal Tissue/Cell Genomic DNA Extraction Kit (Solarbio Science & Technology Co., Ltd., Beijing, China). Mitochondrial NADH1 (*mtND1*) gene was amplified and normalized to nuclear gene control. For heart tissue, the primer sequences were as follows: *mtND1*, 5′-GAGGGAACCAAACTGAACGC-3′ and 5′-TGGATCCGTTCGTAGTTGGAG-3′; *B2M*, 5′-CAGACTCTGCGATGTTTCCA-3′ and 5′-GCCTGAGCACTTCCAGAAAC-3′. For H9c2 cells, the primer sequences were as follows: *mtND1*, 5′-TCCAACTAATAAGCGGCTCCTTCTC-3′ and 5′-CGGCTATGAAGCCTAGGGCGAATGG-3′; *β-actin*, 5′-TGCTATGTTGCCCTAGACTTCG-3′ and 5′-GTTGGCATAGAGGTCTTTACGG-3′.

### 4.16. Western Blotting

Tissues or cells were lysed in RIPA lysis buffer and subjected to SDS-PAGE/Western blotting as previously described [21]. The primary antibodies used were anti-ABAT (Ab108249, Abcam, Cambridge, UK), anti-GAPDH (10494-1-AP, Proteintech, Wuhan, China), anti-β-actin (AF7018, Affinity, Changzhou, China), anti-Cleaved-Caspase3 (9662S, CST, Danvers, MA, USA), anti- PINK1 (WL04963, Wanleibio, Shenyang, China), anti-Parkin (AF0235, Affinity, Changzhou, China) and anti-LC3B (2775S, CST, Danvers, MA, USA).

### 4.17. Statistical Analysis

Statistical analyses and data plotting were performed using GraphPad Prism 8.0 (GraphPad Software, Boston, MA, USA). Data are presented as mean  ±  standard deviation (mean  ±  SD). For comparisons among multiple groups, two-way analysis of variance (ANOVA) was applied, while comparisons between two groups were performed using Student’s t-test. A significance threshold was set at *p* < 0.05.

## 5. Conclusions

In summary, the present study demonstrates that ABAT expression and activity are significantly downregulated in the myocardium following doxorubicin exposure. Cardiac-specific overexpression of ABAT effectively protects against doxorubicin-induced cardiotoxicity, as evidenced by improved cardiac function, reduced serum myocardial injury markers, and attenuated pathological changes. Mechanistically, ABAT exerts its cardioprotective effects by preserving mitochondrial DNA copy number, enhancing mitochondrial respiratory chain complex activity, restoring cellular energy metabolism, alleviating oxidative stress, and ameliorating mitophagy dysregulation. These findings establish ABAT as a critical regulator of mitochondrial homeostasis in the heart and identify it as a novel endogenous defender against doxorubicin-induced cardiomyopathy. Given that ABAT is downregulated in various malignancies and its overexpression suppresses tumor progression, targeting ABAT may offer a unique therapeutic advantage without compromising antitumor efficacy. Collectively, this study provides new insights into the pathogenesis of doxorubicin-induced cardiotoxicity and highlights ABAT as a promising therapeutic target for its management.

## Figures and Tables

**Figure 1:**
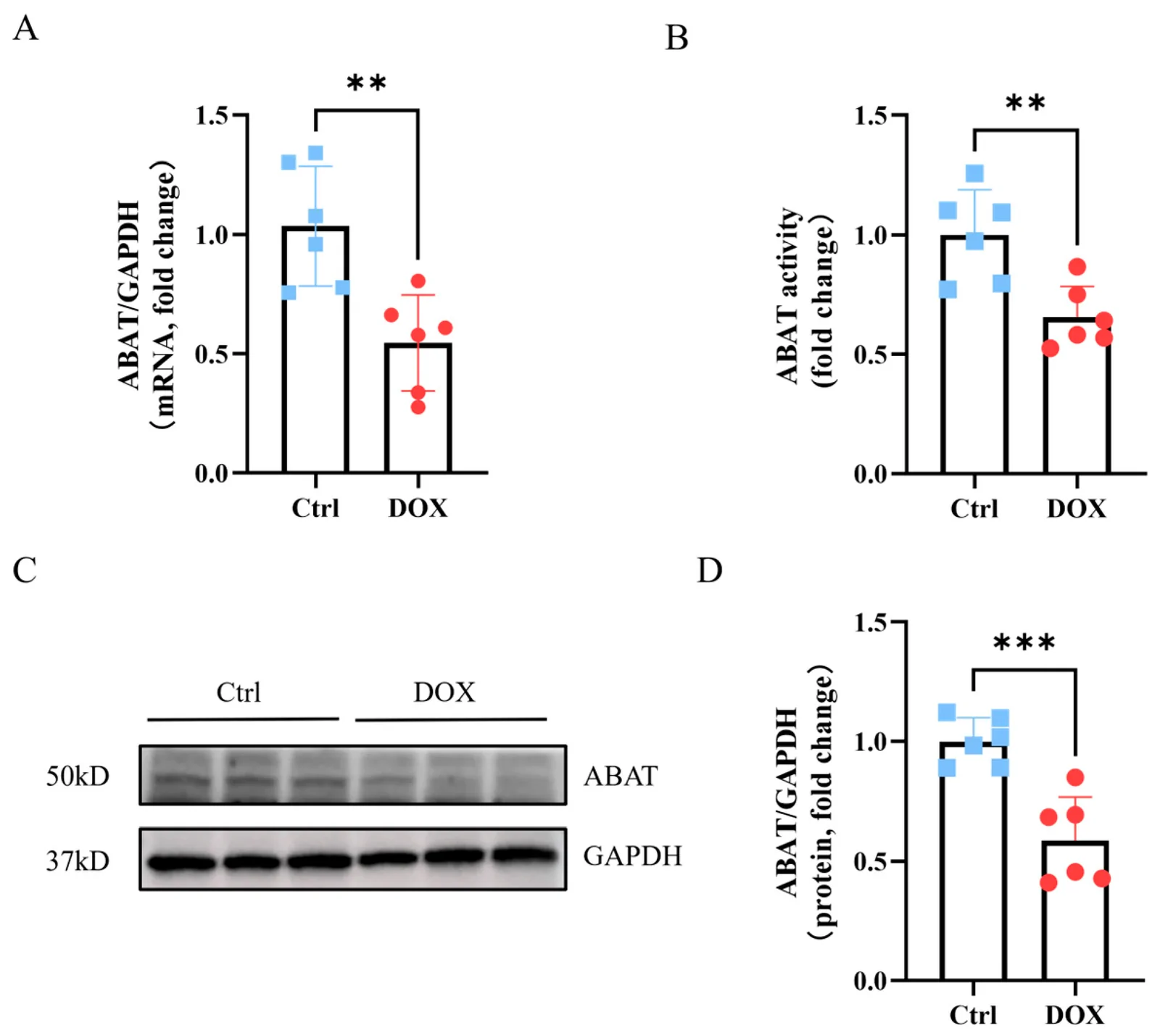
Effects of doxorubicin (DOX) on ABAT level in mouse cardiac tissue. Male C57BL/6 mice were intraperitoneally injected with doxorubicin (15 mg/kg) or saline. Five days later, mice were sacrificed and heart tissue harvested. (**A**) ABAT mRNA levels in the heart were measured by quantitative real-time PCR; (**B**) ABAT enzymatic activity was measured using a colorimetric assay; (**C**) ABAT protein level was detected by Western Blot. (**D**) Quantitative analysis of ABAT protein levels normalized to GAPDH. Ctrl: normal saline control group; DOX: doxorubicin-treated group. Data are presented as mean ± SD (*n* = 6 per group). ** *p* < 0.01, *** *p* < 0.001 vs. Ctrl.

**Figure 2:**
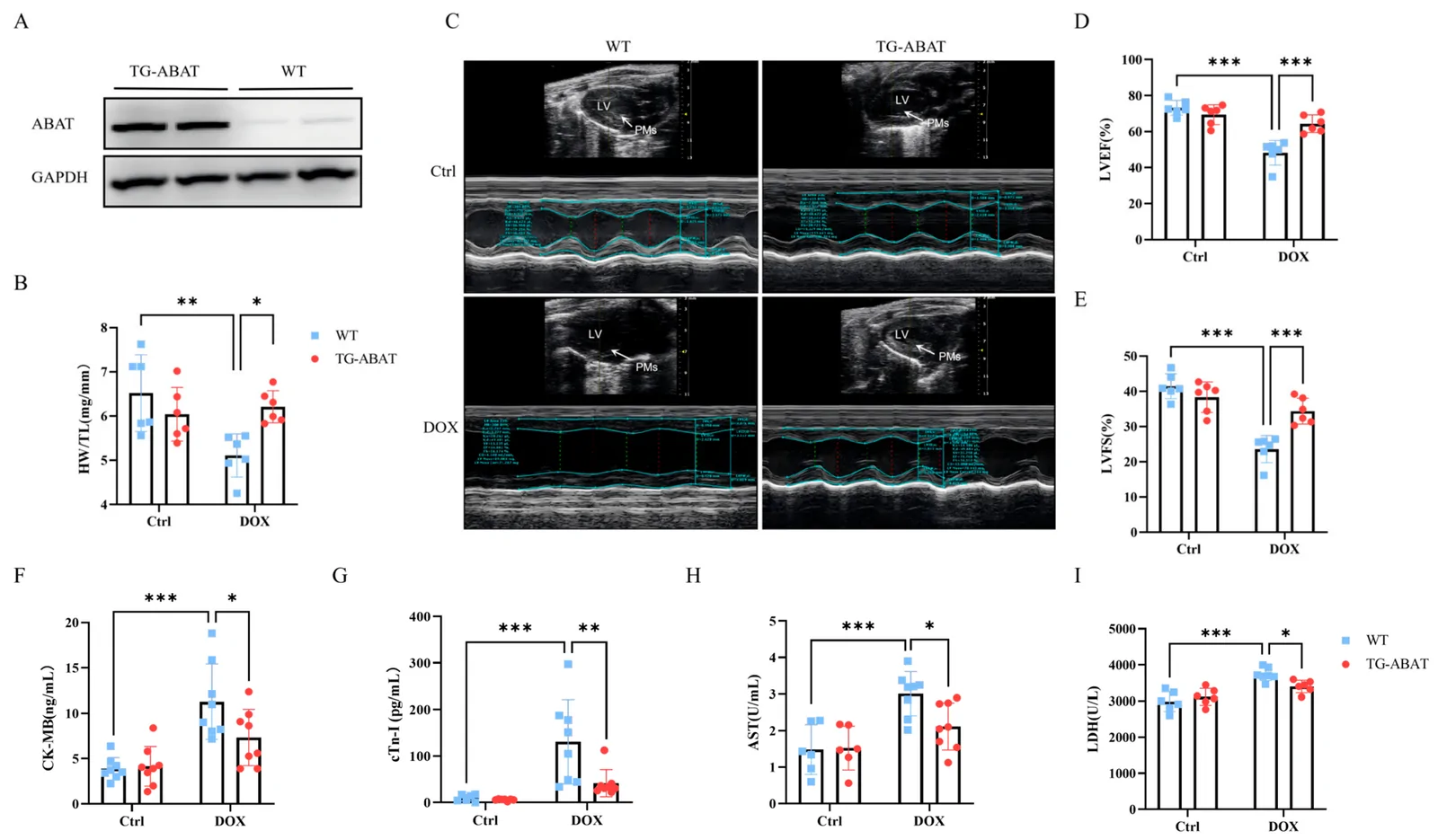
Cardiac-specific overexpression of ABAT ameliorates doxorubicin (DOX)-induced cardiotoxicity in mice. Cardiomyocyte-specific ABAT transgenic (TG-ABAT) mice and wild-type (WT) littermates were intraperitoneally injected with DOX or normal saline (Ctrl). (**A**) Western blot confirming ABAT overexpression in TG-ABAT mouse hearts. GAPDH as loading control; (**B**) Heart weight-to-tibia length ratio (HW/TL); (**C**) Representative parasternal long-axis echocardiographic images showing two-dimensional (B-mode) and M-mode recordings; the left ventricular cavity (LV) and papillary muscles (PMs) are indicated. (**D**) Left Ventricular Ejection Fraction (LVEF) and (**E**) Fractional Shortening (LVFS). Serum levels of (**F**) CK-MB, (**G**) cTn-I, (**H**) AST and (**I**) LDH were measured by colorimetry. Ctrl: normal saline control group; DOX: doxorubicin-treated group; CK-MB: creatine kinase-MB; cTn-I: cardiac troponin I; AST: aspartate aminotransferase; LDH: lactate dehydrogenase. Data are presented as mean ± SD (*n* = 6–8). * *p* < 0.05, ** *p* < 0.01, *** *p* < 0.001.

**Figure 3:**
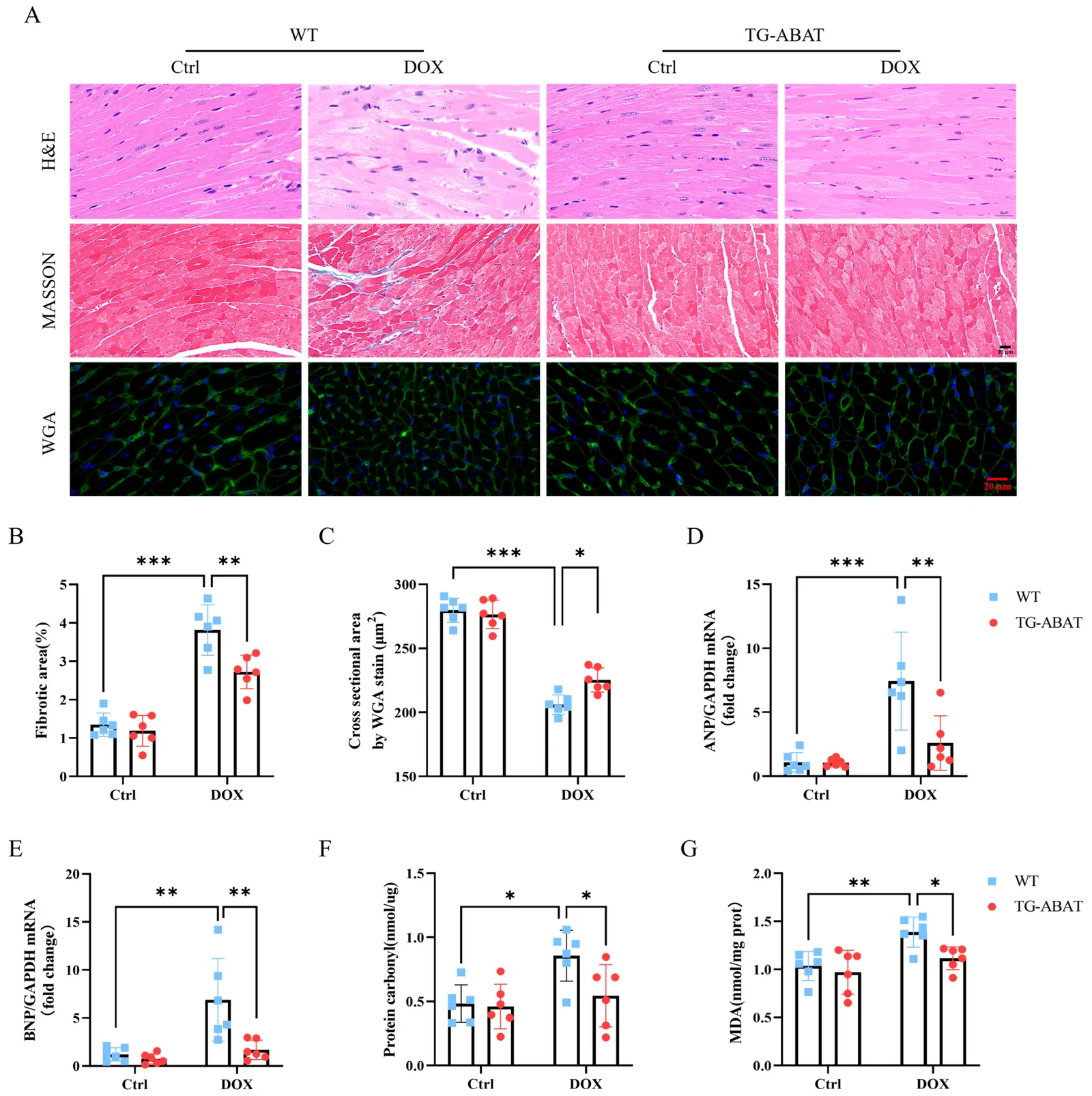
ABAT overexpression ameliorates DOX-induced myocardial structural alterations and oxidative stress in mice. H&E staining was performed to observe myocardial structure, Masson staining to assess myocardial fibrosis, and WGA staining to measure the cross-sectional area of cardiomyocytes. (**A**) Representative images of H&E, Masson, and WGA staining. In the WGA fluorescence images, green indicates the cell membrane stained with WGA, and blue indicates the nuclei stained with DAPI; (**B**) quantitative results of Masson staining; (**C**) quantitative results of WGA staining; (**D**) myocardial *ANP* mRNA levels; (**E**) myocardial *BNP* mRNA levels; (**F**) protein carbonyl level; (**G**) MDA level. WT: wild-type mice; TG-ABAT: cardiomyocyte-specific ABAT overexpression mice; Ctrl: normal saline control group; DOX: doxorubicin-treated group; ANP: atrial natriuretic peptide; BNP: B-type natriuretic peptide; MDA: Malondialdehyde. Data are presented as mean ± SD (*n* = 6), * *p* < 0.05, ** *p* < 0.01, *** *p* < 0.001.

**Figure 4:**
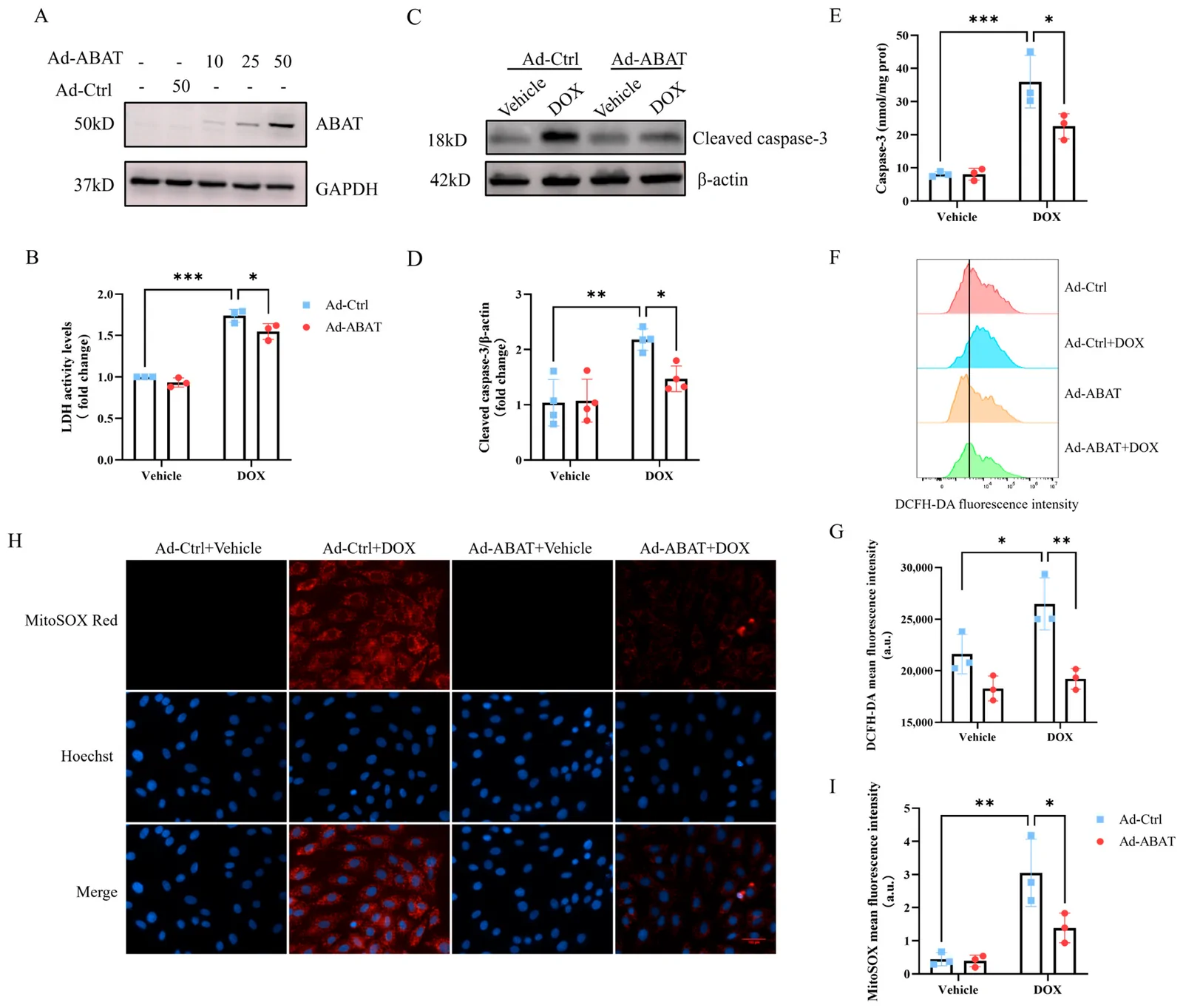
ABAT overexpression alleviates doxorubicin-induced apoptosis and oxidative stress in H9c2 cardiomyocytes. H9c2 cells were infected with empty adenovirus (Ad-Ctrl) or ABAT-overexpressing adenovirus (Ad-ABAT) and then treated with DOX (500 nM) or vehicle for 24 h. (**A**) Western blot analysis of ABAT expression in cells infected with Ad-ABAT at increasing multiplicities of infection (MOI) or Ad-Ctrl; GAPDH served as a loading control. (**B**) LDH activity in the culture medium. (**C**) Representative Western blot of cleaved-caspase-3. (**D**) Quantitative analysis of cleaved-caspase-3 levels. (**E**) Caspase-3 activity measured by colorimetric assay. (**F**) Representative flow cytometric histograms of intracellular ROS levels detected by DCFH-DA probe. (**G**) Quantitative analysis of intracellular DCFH-DA fluorescence intensity. (**H**) Representative fluorescence images of mitochondrial superoxide labeled with MitoSOX Red (red); nuclei were counterstained with Hoechst (blue); merged images are shown; scale bar, 100 μm. (**I**) Quantitative analysis of relative MitoSOX fluorescence intensity. Ad-Ctrl: empty adenovirus; Ad-ABAT: ABAT-overexpressing adenovirus; Vehicle: saline control; DOX: doxorubicin; LDH: lactate dehydrogenase; ROS: reactive oxygen species; MOI: multiplicity of infection. Data are presented as mean ± SD (*n* = 3-4), * *p* < 0.05, ** *p* < 0.01, *** *p* < 0.001.

**Figure 5:**
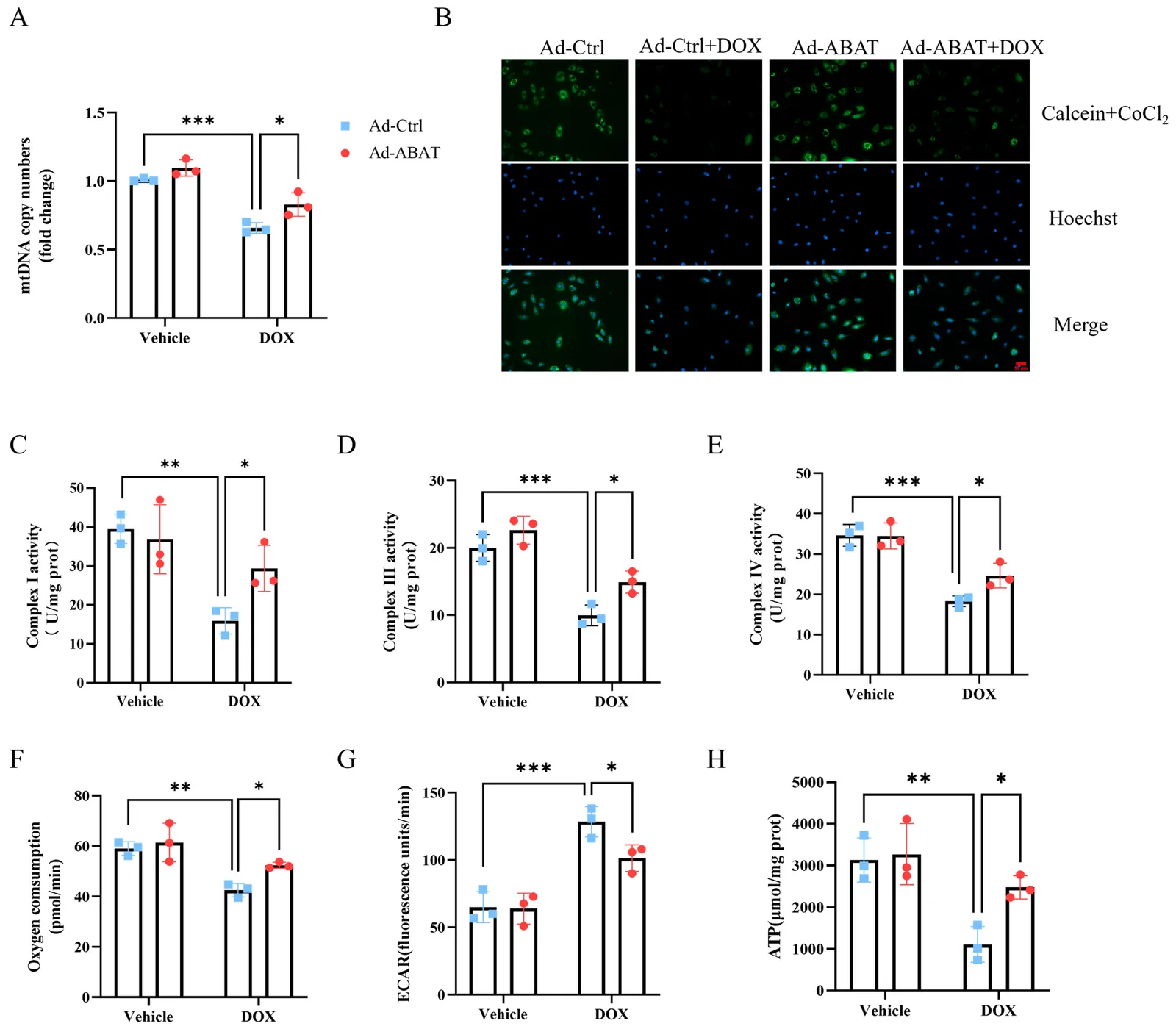
ABAT overexpression preserves mitochondrial integrity and respiratory function in DOX-treated H9c2 cells. (**A**) Mitochondrial DNA (mtDNA) copy number was detected by quantitative PCR; (**B**) MPTP opening assessed by calcein-AM fluorescence quenching assay. Representative calcein fluorescence images of cardiomyocytes from the four indicated groups. Cells were loaded with calcein-AM in the presence of CoCl_2_ to quench cytosolic calcein fluorescence. Under normal conditions, calcein is retained in mitochondria and emits green fluorescence. Upon MPTP opening, calcein leaks into the cytosol and is quenched by CoCl_2_, resulting in loss of mitochondrial fluorescence. Scale bar: 50 μm; (**C**) Mitochondrial respiratory complex I activity; (**D**) Mitochondrial respiratory complex III activity; (**E**) Mitochondrial respiratory complex IV activity; (**F**) oxygen consumption rate (OCR); (**G**) extracellular acidification rate (ECAR); (**H**) ATP levels were measured by colorimetric assays. Ad-Ctrl: empty adenovirus; Ad-ABAT: ABAT-overexpressing adenovirus; Ctrl: normal saline control group; DOX: doxorubicin-treated group; OCR: oxygen consumption rate; ECAR: extracellular acidification rate. Data are presented as mean ± SD (*n* = 3), * *p* < 0.05, ** *p* < 0.01, *** *p* < 0.001.

**Figure 6:**
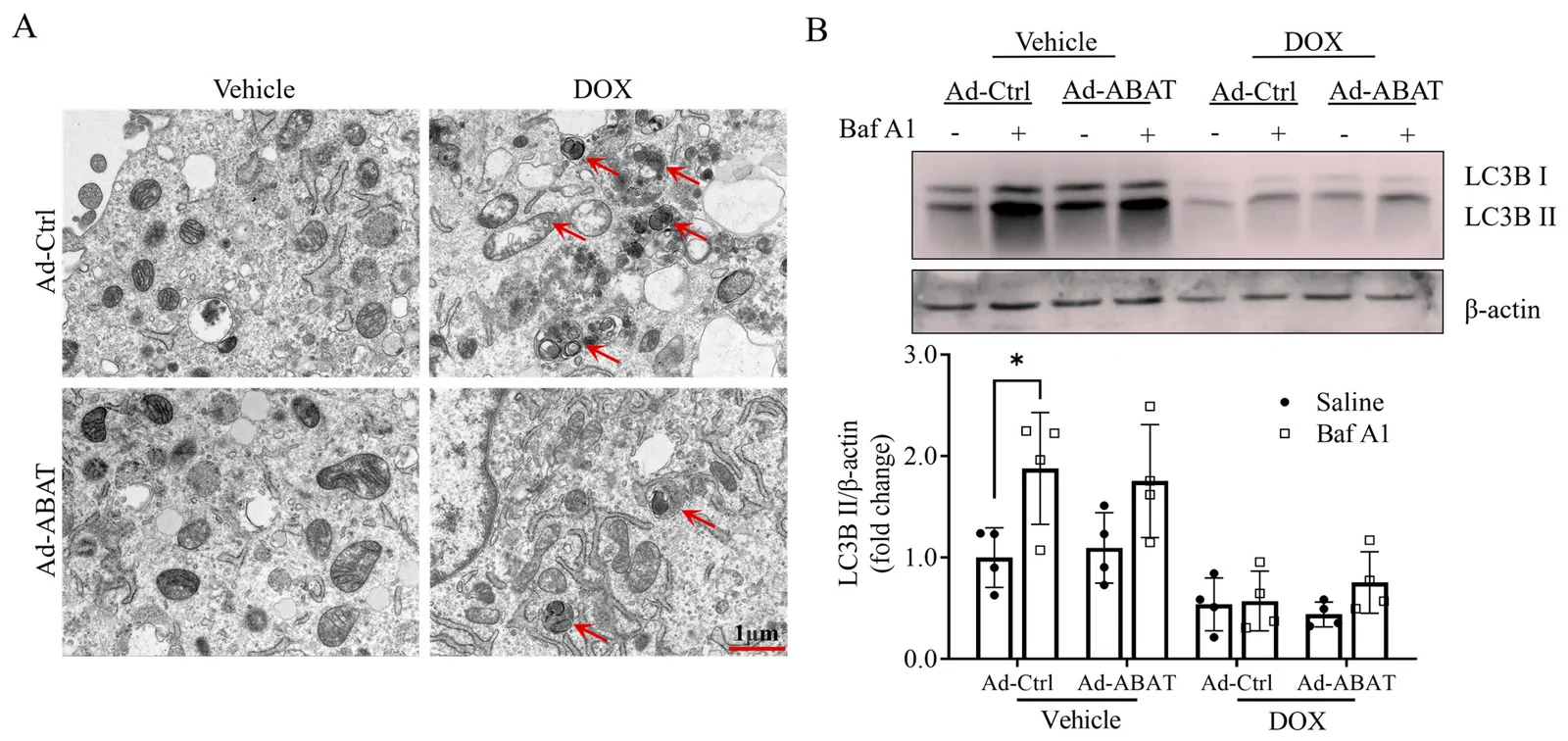

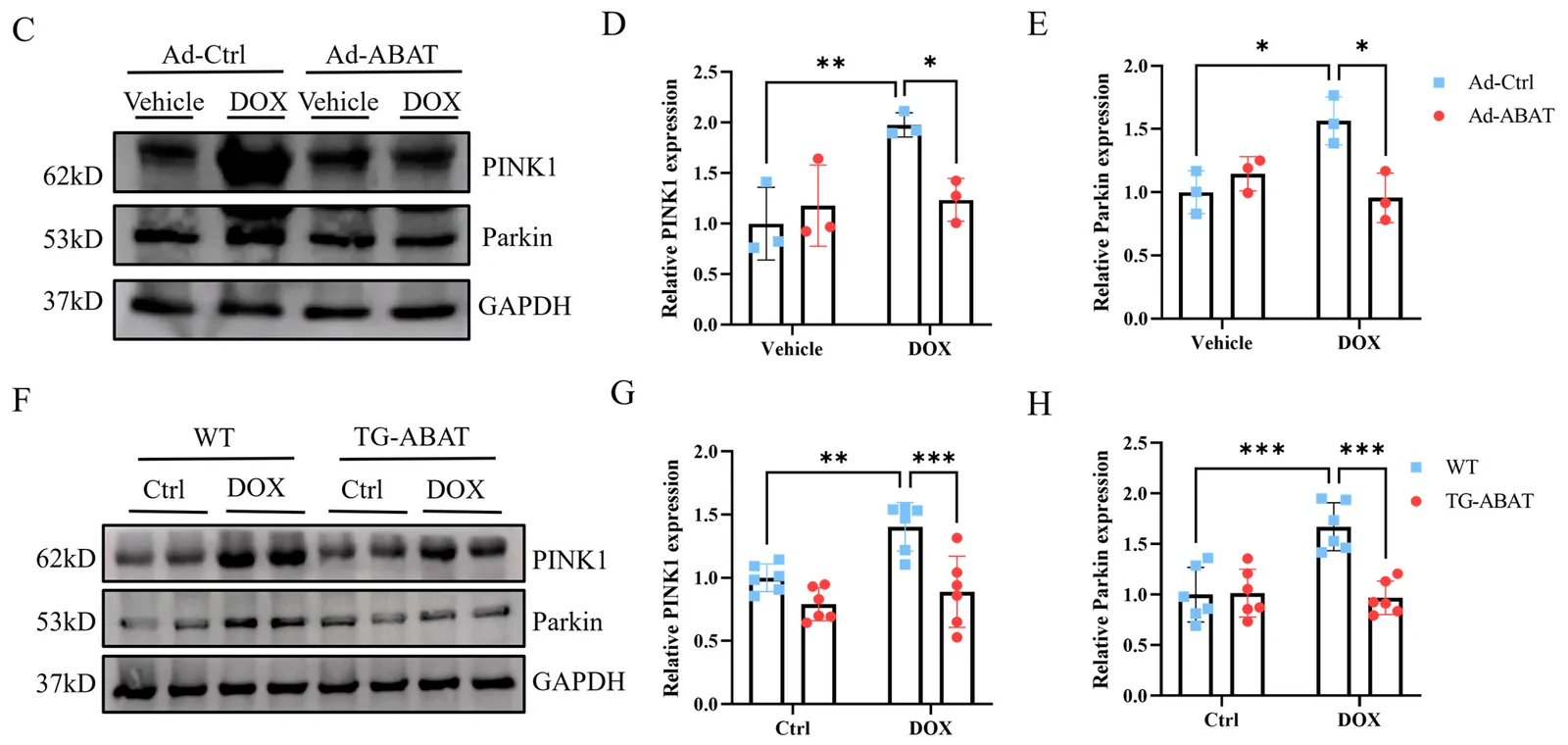
ABAT overexpression preserves mitochondrial ultrastructure and attenuates mitophagy perturbation in DOX-treated H9c2 cells and mouse hearts. (**A**) Mitochondrial ultrastructure in H9c2 cells was visualized by transmission electron microscopy. Arrows point to double-membrane autophagic structures enclosing mitochondria. (**B**) Autophagic flux assessed by Western blotting of LC3B-I/II in cells treated with or without bafilomycin A1 (Baf A1, 100 nM). (**Upper**) panel: representative Western blots; (**lower**) panel: densitometric quantification of LC3B II/β-actin. (**C**) representative Western blots of PINK1 and Parkin in H9c2 cells, with GAPDH as the loading control, and (**D**,**E**) densitometric quantification of PINK1 and Parkin. Ad-Ctrl: empty adenovirus; Ad-ABAT: ABAT-overexpressing adenovirus; Ctrl: normal saline control group; DOX: doxorubicin-treated group. Data are presented as mean ± SD (*n* = 3–4 independent experiments). (**F**) representative Western blots of PINK1 and Parkin in mouse heart tissues and (**G**,**H**) densitometric quantification of PINK1 and Parkin, respectively. WT: wild-type mice; TG-ABAT: cardiomyocyte-specific ABAT overexpression mice; Ctrl: normal saline control group; DOX: doxorubicin-treated group. Data are presented as mean ± SD (*n* = 6 mice per group), * *p* < 0.05, ** *p* < 0.01, *** *p* < 0.001.

## Data Availability

All data supporting the findings of this study are available from the corresponding author upon reasonable request.

## Supplementary Materials

The following supporting information can be downloaded at: https://www.mdpi.com/article/10.3390/ijms27219994/s1.
