# Supplementary material for: ABAT Overexpression Protects Against Doxorubicin-Induced Cardiotoxicity by Preserving Mitochondrial Homeostasis
Source: Int J Mol Sci. 2026 Nov 7;27(21):9994. doi: 10.3390/ijms27219994 (PMC13764394; doi:10.3390/ijms27219994)
Supplement: Supplementary file 1 [file ijms-27-09994-s001.zip › ijms-4628962-supplementary.pdf]

Supplementary Materials

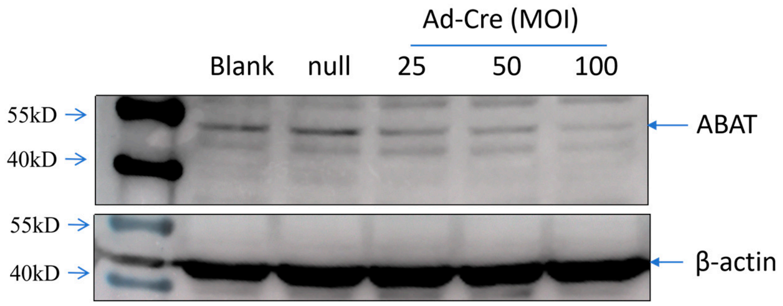

Figure S1. Validation of the ABAT band by adenoviral Cre-mediated knockdown in ABAT-floxed neonatal mouse cardiomyocytes. Primary neonatal cardiomyocytes isolated from ABAT-floxed mice were infected with an adenovirus encoding Cre recombinase (Ad-Cre) at multiplicities of infection (MOI) of 25, 50, or 100. The ~50 kDa band detected by the anti-ABAT antibody (upper panel) was progressively reduced with increasing Ad-Cre dose in a dose-dependent manner, while the loading control β-actin (lower panel) was unchanged. This genetic loss-of-function experiment independently confirms that the band assigned to ABAT by its molecular weight throughout the manuscript indeed represents ABAT.

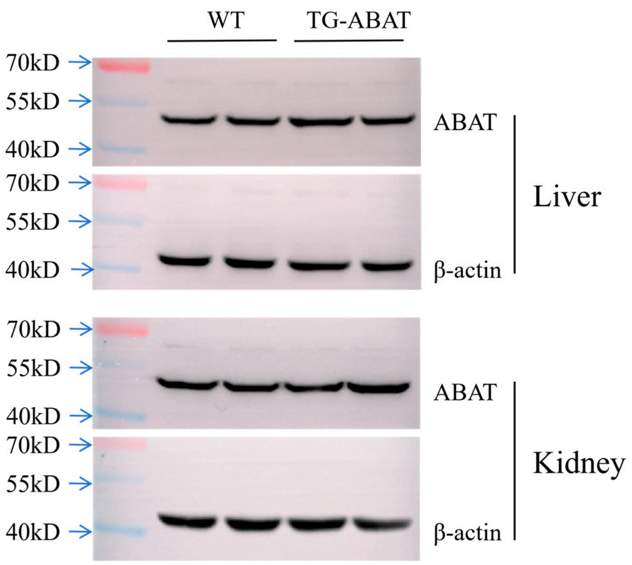

Figure S2. ABAT protein expression in liver and kidney tissues. Liver and kidney tissues from transgenic mice with cardiomyocyte-specific ABAT over-expression (TG-ABAT) and their littermate controls (WT) were collected and subjected to western blot analysis. Representative western blots for ABAT and β-actin show that the protein levels of ABAT do not differ between TG-ABAT mice and WT mice.

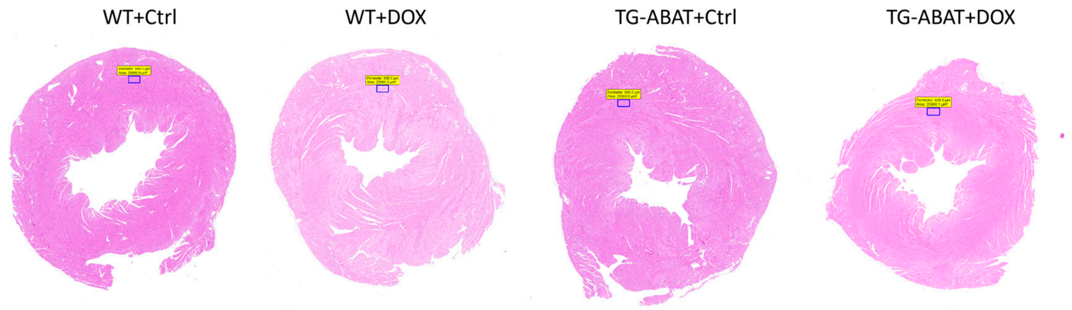

Figure S3. Panoramic overview of H&E-stained myocardial sections. Representative low-magnification (2.5×) images of hematoxylin and eosin (H&E)-stained heart sections. Blue boxes indicate the selected regions of interest (each with a perimeter of 600 μm), which were subsequently magnified at 100× and presented in the main text (Figure 3A) to illustrate detailed cellular morphology.

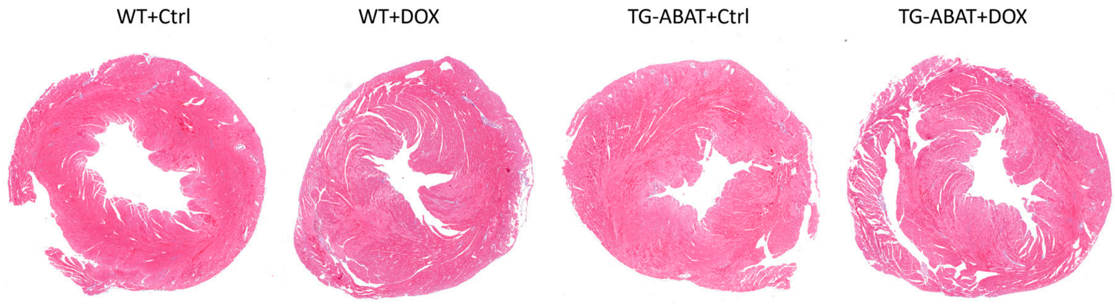

Figure S4. Panoramic overview of Masson's trichrome-stained whole ventricular cross-sections. Representative whole transverse cross-sections of the ventricles from different groups. Masson's trichrome staining was used to evaluate overall collagen deposition (blue) and myocardial architecture (red).

Table S1. Echocardiographic analysis in mice

	WT+Ctrl	WT+DOX	TG-ABAT+Ctrl	TG-ABAT+DOX
Heart Rate（bpm）	423.67±38.66	371.5±27.63*	427±25.27	410.83±21.64
EF (%)	73.22±4.12	48.24±6.86*	69.36±5.52	64.36±4.91$^{\#}$
FS (%)	41.48±3.55	23.62±3.85*	38.36±4.32	34.4±3.67$^{\#}$

FS, fractional shortening; EF, ejection fraction; WT, wild-type; TG-ABAT, cardiomyocyte-specific ABAT transgenic; Ctrl, normal saline control; DOX, doxorubicin. Data are mean ± SD (n = 6). $^{*}P<0.05$ *vs.* Ctrl+WT, $^{\#}P<0.05$ *vs.* DOX+WT.
